# A systems analysis of structures, dynamics, archetypes of the Somalia famine, 2011–2012

**DOI:** 10.1057/s41271-026-00658-1

**Published:** 2026-08-12

**Authors:** Udita Sanga

**Affiliations:** Friedman School of Nutrition Science & Policy, Tufts University, Medford, USA

**Keywords:** Famines, Somalia, Causal loop diagrams, Systems mapping, Archetypes

## Abstract

The 2011–2012 Somalia famine was a complex humanitarian disaster shaped by interacting environmental, economic, social, and political factors. This paper applies a qualitative systems analysis to examine famine structure, dynamics, and archetypes to demonstrate how systems modelling can deepen understanding of famine as a complex system. The study addresses three questions: (1) what key variables and feedback dynamics created pressures and constraints within the Somalia famine system; (2) how system components interacted to produce outcomes such as food insecurity, malnutrition, and mortality; and (3) which system archetypes characterized system behaviour. Using a causal loop diagram (CLD), the study maps interdependencies across environmental, biophysical, economic, social, health, political, and institutional domains within the famine system. The CLD illustrates the specific reinforcing feedbacks that acted as pressures, constraining feedbacks that acted as holds and balancing feedbacks that acted as stabilizers of the system. We observe three key system archetypes: Escalation, Fixes that fail and Shifting the burden. Overall, the study demonstrates that famine emerges from dynamic system interactions rather than isolated drivers, highlighting the value of systems thinking for improving explanation, policy insight, and intervention design in complex humanitarian crises.

**Supplementary Information:**

The online version contains supplementary material available at 10.1057/s41271-026-00658-1.

## Key messages

- Systems analysis clarifies complex interactions, revealing why rapid shifts in malnutrition and mortality occur.
- Reinforcing feedbacks act as pressures, constraining dynamics act as holds, and balancing feedbacks help stabilize the system, jointly shaping outcomes such as food insecurity, malnutrition, and mortality.
- It helps identify factors that create pressure, hold and rebalance, and lead to famine archetypes like Escalation, Fixes that fail, and Shifting the burden.

## Introduction

Famines are complex, multidimensional crises that arise from the interaction of natural, environmental, economic, and political factors. These interconnected drivers disrupt food production, distribution, and access, leading to severe food insecurity accompanied by rapid increases in malnutrition and mortality among affected populations. Famines have been conceptualized in several different ways: as an event, a process and a system [1–3]. The event-based conceptualization views famines as a discrete phenomenon that afflicts a population due to lack of sufficient food as well as entitlements that enable people to access food, leading to high malnutrition and mortality rates [3]. Devereux [1] conceptualized famines as long-term social and historical processes that operate over time to create and perpetuate malnutrition and mortality. Howe [4], on the other hand, proposes a composite perspective of famines where “famines are *events* that result from *processes*”. Howe describes the dynamic evolution of famines from formation to collapse when *pressure* (a combination of disruptive factors) is kept in place by a *hold* leading to *self-reinforcing dynamics* that tip over into a *famine system* before *rebalancing* [5]. From a systems perspective, famine can be seen as a waxing and waning of critical dynamics that shape how a famine progresses over time, creating recurring patterns. Howe [4] terms these recurrent patterns as ‘famine archetypes’ such as rapid food price increases (price spiral), heavy reliance on international aid (aid magnet), intense media coverage (media frenzy), a population exceeding the environment’s capacity to support it (overshoot) and reaching the most critical point of crisis (peaks), marked by widespread malnutrition and mortality. These archetypes are similar but conceptually different to the well-known ‘system archetypes’ identified by Kim and Lannon [6] and Wolstenholme [7], including Limits to Growth, Shifting the Burden, Fixes that Fail, Growth and Underinvestment, Eroding Goals, Escalation, Drift to Extinction, and Tragedy of the Commons.

**Fig. 1:**
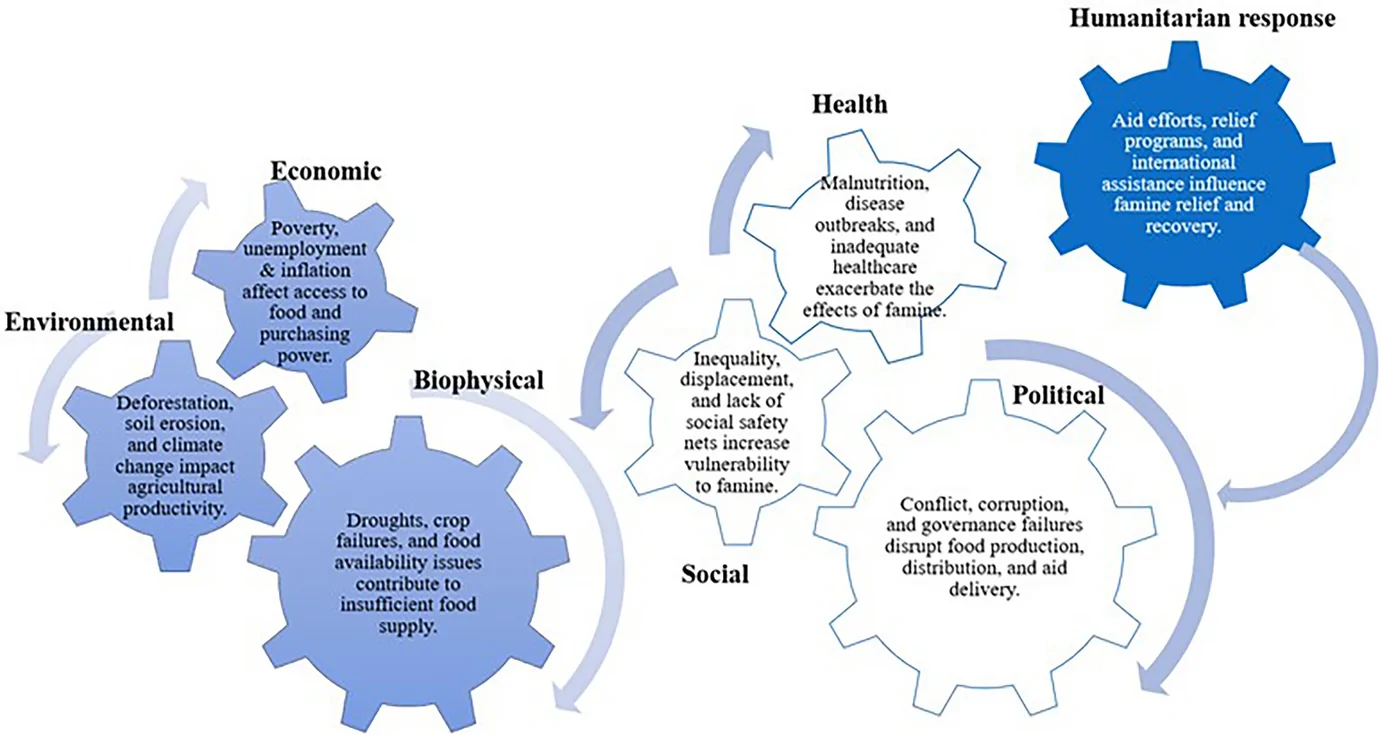
Famine as a complex system with interlinked environmental, biophysical, economic, social, health, political, and humanitarian dimensions

How we conceptualize a famine has implications for how it is analysed and addressed. Viewing famine as an event undertakes a static approach, where solutions focus on recovery, emphasizing bouncing back from the crisis. However, systems analysis offers a complementary perspective by examining the relationships among system components and the ways in which these interactions reinforce or counteract one another over time to create conditions for a famine [4]. This perspective moves beyond linear cause-and-effect explanations and provides a *dynamic* understanding of famine as an emergent outcome of a complex adaptive system. Fortnam and Hailey [3] emphasize the importance of understanding famine as a complex system in which multiple interacting social-ecological drivers shape famine dynamics and outcomes. These interconnected drivers are illustrated in Fig. 1, which depicts famine as a complex system comprising linked environmental, biophysical, economic, social, health, political, and humanitarian dimensions. These factors interact dynamically through feedback structures rather than operating in a simple linear sequence, making it difficult to understand or address through a single-factor explanation. Systems theory and tools can help uncover these essential dynamics within famine systems and shift attention to the broader structural and causal factors at play. This allows us to reorient our understanding of resilient solutions that allow the system to persist while maintaining essential structure [8]. The objective of this paper is to demonstrate how systems modelling and analysis can contribute to a deeper understanding of famine as a complex system through an examination of the 2011–2012 Somalia famine. Specifically, the paper addresses three key questions: (1) What were the key variables and feedback dynamics that acted as pressures and constraints within the Somalia famine system? (2) How did the various components of the system interact to produce critical outcomes such as food insecurity, malnutrition, and mortality? and (3) What system archetypes characterized the behaviour of the Somalia famine system? By addressing these questions, the paper illustrates the value of systems analysis for understanding the complex interactions, feedback processes, and structural conditions that shape famine dynamics and outcomes.

## Methodology

We used casual loop diagrams (CLDs) as visual tools for systems thinking and system dynamics analysis to represent feedback loops and causal relationships between system variables, offering insights into how these variables interact over time to shape system behaviour. A literature review of the 2011–2012 Somalia famine enabled the identification of the key environmental, economic, social and political variables that influenced famine outcomes. Variables were selected based on their prominence in the literature and their contribution to famine dynamics. These variables were then connected through causal loop diagrams that are composed of several fundamental elements, including variables (or nodes), which represent factors within the system that change over time; causal links, represented by directional arrows that indicate how changes in one variable influence another; and polarity, which is assigned to each causal link to indicate whether variables move in the same direction (positive relationship) or in opposite directions (negative relationship). These relationships combine to form feedback loops, which are closed chains of causality that capture cyclical processes within the system. Reinforcing feedback loops amplify change and can lead to accelerating growth or decline, whereas balancing feedback loops counteract change and promote stability. For each pair of variables, the direction of influence was identified and represented using causal links. Positive (+) links indicate that an increase (or decrease) in one variable leads to a corresponding increase (or decrease) in another variable, while negative (–) links indicate that an increase in one variable leads to a decrease in the other, or vice versa. By tracing causal pathways and feedback processes, the analysis explored how interactions among the variables generated famine outcomes such as mortality and food insecurity. The CLDs were further analysed to identify system archetypes that characterized the behaviour of the Somalia famine system. System archetypes are recurring feedback structures that produce characteristic patterns of behaviour across a wide range of systems. They provide a framework for understanding the underlying mechanisms driving system performance and for identifying potential intervention points. Following the approach of Kim and Lannon [6], the CLD was examined for evidence of well-established archetypes, each associated with combinations of reinforcing and balancing feedback loops, delays, unintended consequences, and threshold effects.

## Overview: 2011–2012 Somalia famine

The 2011–2012 Somalia famine is among the most extensively studied famine case studies and is widely regarded as one of the most severe and complex humanitarian disasters in contemporary history. The literature clearly demonstrates how intersecting ecological, political, economic, and social dynamics contributed to both the emergence and persistence of the famine. Rather than being caused by a single event, the famine was driven by several disruptive factors including severe droughts linked to the La Niña phenomenon, a sudden rise in global food prices, and ongoing conflict and geopolitical insecurity in the country (see [9–12]). The country’s social structure is predominantly clan-based, with clan affiliations significantly influencing resource management and shaping agricultural and livelihood strategies [10]. The livelihoods of pastoralists and agro-pastoralists are highly vulnerable to climate variability, droughts, and extreme weather events, compounded by rapid deforestation, severe soil erosion, and overgrazing [13, 14]. These challenges are further intensified by inadequate infrastructure which hinders agricultural productivity and perpetuates poverty [15]. These interconnected factors contribute to Somalia’s heightened susceptibility to drought-induced food insecurity and famine.

The onset of the famine in 2011–2012 famine was marked by the initial emergence of severe drought conditions across the Horn of Africa. Drought, triggered by the failure of the *deyr* rains in late 2010 and the *gu* rains in early 2011, severely impacted the agricultural cycle, leading to drastically reduced harvests, dried-up water sources, and sharp decline in livestock viability [16]. These harsh conditions made survival exceedingly difficult for agro-pastoral populations, particularly in the southern regions of Somalia. Simultaneously, the dramatic increase in global food prices in early 2011 further strained the ability of affected populations to secure food, compounding the already dire effects of the drought. The escalating crisis culminated in the United Nations officially declaring a famine in two southern regions of Somalia, Bakool and Lower Shabelle, in July 2011. The transitional government, supported by Western and East African nations, engaged in a counterinsurgency against Al-Shabab as part of the broader ‘War on Terror’. The conflict complicated humanitarian efforts, as Al Shabab, controlling significant portions of southern Somalia, imposed restrictions on aid delivery, while Western donors, wary of inadvertently supporting the insurgency, enacted counter-terrorism legislation that deterred humanitarian agencies from providing aid [17]. The protracted conflict between the Somali government, its allies, and the insurgent group Al Shabab exacerbated the situation, leading to widespread insecurity and displacement. The famine expanded to additional regions, including Middle Shabelle, Afgoye, and the Internally Displaced Persons (IDP) settlements in Mogadishu. By December 2011 and January 2012, the situation in some areas began to improve, largely due to sustained humanitarian assistance and the return of seasonal rains. The famine system in Somalia began to rebalance by mid-2012, but the toll was catastrophic. By the time the famine subsided, an estimated 258,000 people had died, nearly half of whom were children under the age of five [18]. On 3 February 2012, the United Nations declared that famine conditions had ended in Somalia, signalling the conclusion of the acute famine phase. However, this did not mean the crisis was fully resolved. Many regions continued to face severe food insecurity and malnutrition, requiring ongoing support. Throughout 2012, the focus shifted to recovery and rehabilitation efforts, including rebuilding agricultural infrastructure, providing long-term food security solutions, and supporting displaced populations. This phase represents the gradual stabilization and recovery from the famine.

## Results and discussion

### Systems mapping of Somalia famine

**Fig. 2:**
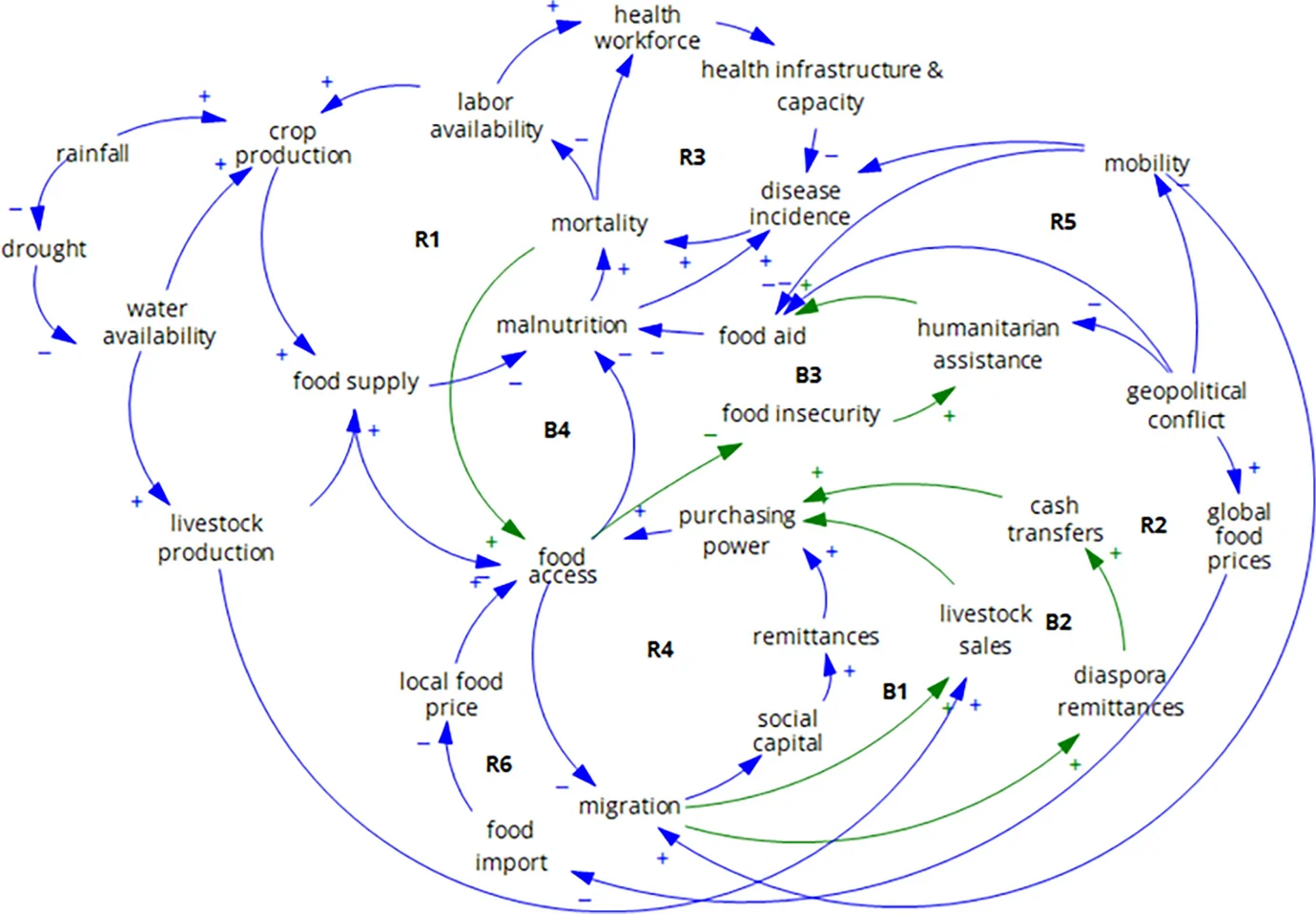
Integrated causal loop diagram (CLD) of famine dynamics showing reinforcing (R) and balancing (B) feedback loops. The diagram illustrates how interacting social, ecological, economic, and political processes generate systemic pressure leading to food insecurity and increased mortality. Reinforcing loops (R1–R6) (in blue) amplify famine outcomes. Balancing loops (B1–B4) (in green) represent coping and stabilizing mechanisms

The causal loop diagram (Fig. 2) translates the narrative presented above into a systems map of the 2011–2012 Somalia famine. The diagram highlights the interconnected ecological, social, economic, and political processes that shaped famine dynamics, revealing how drought, rainfall variability, water scarcity, crop failure, and livestock losses interacted with conflict, displacement, humanitarian assistance, remittances, and social capital. By making these relationships explicit, the CLD illustrates the feedback structures through which environmental shocks were amplified by social and political conditions, ultimately affecting food availability, food access, malnutrition, and mortality. At the same time, it highlights the humanitarian and social support mechanisms such as food aid, cash transfers, remittances, and social capital that acted to mitigate rising mortality and stabilize the system, although their effectiveness was often constrained by conflict, governance challenges, and restrictions on aid delivery.

The analysis identifies the key reinforcing feedback loops that generated pressure within the famine system and created holds by constraining its capacity to recover as well as the balancing feedback loops that enabled the system to rebalance. The Somalia famine system was driven by six interconnected reinforcing feedback loops (R1–R6) that collectively amplify food insecurity and mortality (see Supplementary Material Table 1M). The Climate–Agriculture–Mortality loop (R1) shows how drought reduced agricultural and livestock productivity, lowering food availability and increasing malnutrition and mortality, which further weakened labour capacity and reduced production. The Global Price Shock and Import Dependency loop (R2) demonstrates how rising global food prices reduced imports and increased local prices, limiting food access and worsening malnutrition and mortality, with broader geopolitical instability further reinforcing these pressures. The Health System Collapse loop (R3) captures how weak healthcare infrastructure increased disease incidence and mortality, which in turn further degraded health system capacity. The Migration–Remittance–Food Access loop (R4) shows how food insecurity drove migration, reducing social capital and remittances, thereby lowering household purchasing power and further restricting food access. The Conflict–Aid Disruption loop (R5) highlights how conflict and governance constraints disrupted humanitarian assistance through aid diversion and restricted mobility, increasing malnutrition and mortality and reinforcing instability. Finally, the Mobility and Access Constraint loop (R6) illustrates how conflict restricted movement and migration, limiting both access to food and coping options, thereby further deepening malnutrition and preventing system recovery.

The Somalia famine system included four key balancing feedback loops that attempted to counteract and rebalance rising food insecurity and mortality, although their effectiveness was often limited (see Supplementary Material Table 2). The Migration and Asset Liquidation loop (B1) operated when households responded to reduced food access by migrating or selling livestock, temporarily increasing purchasing power and providing short-term relief from food insecurity, but without addressing underlying vulnerabilities. The Humanitarian Food Aid loop (B2) was intended to stabilize the system by increasing food aid, thereby improving food availability and reducing malnutrition and mortality; however, its impact was significantly constrained by conflict and restrictions such as Al-Shabaab activity and OFAC regulations. The Informal Financial Support loop (B3) functioned through diaspora remittances and hawala-based cash transfers, which increased household purchasing power and improved food access, offering a partial but uneven stabilizing effect on nutrition outcomes. Finally, the Environmental and Market Recovery loop (B4) reflect exogenous improvements such as increased rainfall and lower global food prices, which enhanced food availability, reduced prices, and improved access to food, leading to decline in malnutrition and mortality.

### System archetypes

**Fig. 3:**
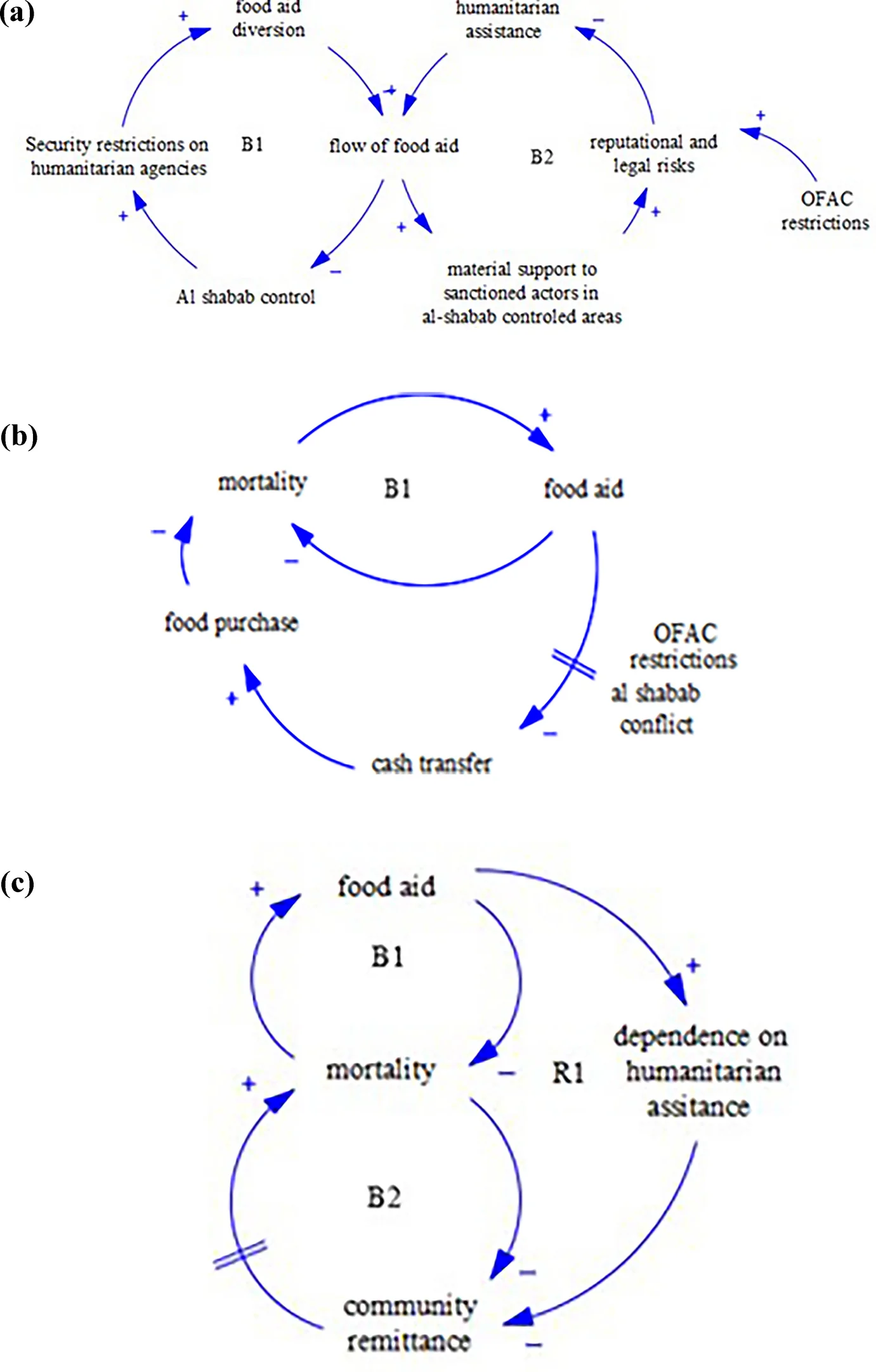
System archetypes in Somalia. **a** Escalation: a situation where two or more entities engage in actions that intensify in response to each other. **b** Fixes that fail: a situation where a solution is applied to a problem, providing temporary relief but ultimately leading to unintended consequences that exacerbate the original issue. **c** Shifting the burden: a situation where a problem is addressed with a short-term solution that alleviates symptoms but fails to address the underlying issue

Three types of system archetypes were found within the system. First, Escalation: a situation where two or more entities engage in actions that intensify in response to each other (Fig. 3a) The CLD highlights two balancing loops (B1 and B2) in which actions taken by different actors inadvertently decrease flow of food aid. In B1, Al Shabab increases restrictions on humanitarian agencies to strengthen control over food aid, reducing aid flows to affected populations and worsening food insecurity. In B2, OFAC sanctions increase the legal and reputational risks faced by humanitarian organizations, leading to reduced humanitarian assistance and further decline in flow of food aid. These dynamics create a vicious cycle in which attempts by both Al Shabab and external actors to control aid flows unintentionally intensify food insecurity, reduce the effectiveness of humanitarian response, and deepen the famine. Second, Fixes that fail: a situation where a solution is applied to a problem, providing temporary relief but ultimately leading to unintended consequences that exacerbate the original issue (Fig. 3b). The causal loop diagram illustrates a balancing feedback loop (B1) in which food aid initially reduces famine mortality. However, delays caused by OFAC restrictions and conflict with Al Shabab reduce the flow of food aid over time, increasing reliance on cash transfers. Cash transfers enable households to purchase food, helping to lower mortality rates despite declining aid. Third, Shifting the burden: a situation where a problem is addressed with a short-term solution that alleviates symptoms but fails to address the underlying issue (Fig. 3c). The causal loop diagram illustrates two balancing loops (B1 and B2) and one reinforcing loop (R1). In the short term, increased food aid reduces mortality (B1), while community remittances also help lower mortality when humanitarian assistance is constrained (B2). However, food aid increases dependence on humanitarian assistance, creating a reinforcing loop (R1) that strengthens reliance on external support. As dependence grows, long-term resilience is weakened, leaving underlying drivers of food insecurity unresolved.

## Future directions for model development

This study relied on a qualitative causal loop diagram (CLD) to represent the complex dynamics of famine systems. While this approach is effective for mapping relationships and identifying reinforcing and balancing feedback structures, it also points to important avenues for further development. First, the CLDs provide a high-level representation of a highly heterogeneous and spatially differentiated system by aggregating diverse populations, institutions, and geographic regions within Somalia into single variables. This aggregation is useful for identifying overarching system structures and feedback relationships; however, it necessarily smooths over important local variations in vulnerability, resilience, and response capacity that shape how famine dynamics are experienced in different contexts. Second, the CLD represents a static structural snapshot of the system, capturing relationships at a conceptual level rather than simulating their evolution over time. As such, it does not explicitly represent temporal dynamics such as delays, accumulation processes, or changes in feedback strength, which are critical for understanding how famine conditions unfold and intensify over time. This also means that the model does not yet support empirically testable forecasts of famine progression or the identification of precise transition thresholds between stability, crisis, and recovery. Third, the current CLD does not enable systematic sensitivity analysis or scenario testing to assess how variations in key drivers such as humanitarian aid flows, mobility restrictions, and market shocks quantitatively influence system outcomes. Future research could extend this work by translating the CLD into a formal system dynamics model. This would enable quantitative simulation, sensitivity analysis, and scenario testing under varying levels of conflict, humanitarian access, migration, and climatic conditions. Such an extension would strengthen the ability to identify critical thresholds, evaluate policy interventions, and explore how small changes in system parameters may shift outcomes from reinforcing crisis dynamics towards stabilization and recovery.

## Conclusions

The systems analysis presented in this study builds on established understandings of famine processes while advancing a systems perspective that highlights the interdependence between social, ecological, economic, political, and humanitarian dynamics. The causal loop diagram (CLD), like all systems maps, is a deliberate abstraction of reality. Rather than fully representing complexity, it functions as an analytical tool for revealing how system behaviour emerges from interactions among components and feedback processes. This approach enables the identification of key elements of the famine system across environmental, biophysical, economic, social, health, political, and institutional domains, and makes explicit the relationships between them. In doing so, it shows how reinforcing pressures and constraining dynamics interact over time to shape famine outcomes, including food insecurity, malnutrition, and mortality. It also supports the identification of recurring system archetypes and behavioural patterns that explain how seemingly distinct processes converge to produce these outcomes. Beyond description, systems mapping provides a structured framework for managing complexity. It clarifies system boundaries, defines the spatial and temporal scope of analysis, and ensures an appropriate level of abstraction. As a conceptual model, it captures the system’s feedback architecture, showing how components reinforce or counteract one another over time, and distils a complex, multi-dimensional phenomenon into a coherent and interpretable representation. The mapping process also supports theory building and policy analysis by enabling the formulation of hypotheses about system behaviour, the identification of potential leverage points, and the exploration of alternative intervention strategies. Overall, this systems approach shifts the analytical focus from isolated drivers of famine to the dynamic interactions that generate, sustain, or mitigate it, thereby strengthening both explanatory insight and policy relevance.

## Supplementary Information

Below is the link to the electronic supplementary material.

Supplementary file1 (DOCX 18 KB)

## Data Availability

No datasets were generated or analysed during the current study.

## References

[1] Devereux S. Sen’s entitlement approach: critiques and counter-critiques. Oxf Dev Stud. 2001;29(3):245–63.

[2] Quak E. The drivers of acute food insecurity and the risk of famine. The Institute of Development Studies and Partner Organisations. Report. 2021; https://hdl.handle.net/20.500.12413/16950.

[3] Fortnam M, Hailey P. Insights from social-ecological systems thinking for understanding and preventing famine. Disasters. 2024;48: e12621. 10.1111/disa.12621. 38441338 10.1111/disa.12621

[4] Howe P. Archetypes of famine and response. Disasters. 2010;34(1):30–54. 19624703 10.1111/j.1467-7717.2009.01113.x

[5] Howe P. Famine systems: a new model for understanding the development of famines. World Dev. 2018;105:144–55.

[6] Kim DH, Lannon C. Applying systems archetypes. Waltham: Pegasus Communications; 1997.

[7] Wolstenholme EF. Towards the definition and use of a core set of archetypal structures in system dynamics. Syst Dyn Rev. 2003;19(1):7–26.

[8] Holling CS. Engineering resilience versus ecological resilience. Eng Ecol Constr. 1996;31(1996):32.

[9] Haan N, Devereux S, Maxwell D. Global implications of Somalia 2011 for famine prevention, mitigation and response. Glob Food Sec. 2012;1(1):74–9.

[10] Majid N, McDowell S. Hidden dimensions of the Somalia famine. Glob Food Sec. 2012;1(1):36–42.

[11] Maxwell D, Fitzpatrick M. The 2011 Somalia famine: Context, causes, and complications. Glob Food Sec. 2012;1(1):5–12.

[12] Hillbruner C, Moloney G. When early warning is not enough—Lessons learned from the 2011 Somalia Famine. Glob Food Secur. 2012;1(1):20–8.

[13] Lwanga-Ntale C, Owino BO. Understanding vulnerability and resilience in Somalia. Jàmbá: J Disaster Risk Stud. 2020;12(1):1–9. 10.4102/jamba.v12i1.856 PMC7768599 33408804

[14] Salama P, Moloney G, Bilukha OO, Talley L, Maxwell D, Hailey P, Hillbruner C, Masese-Mwirigi L, Odundo E, Golden MH. Famine in Somalia: evidence for a declaration. Glob Food Secur. 2012;1(1):13–9.

[15] Pape UJ, Wollburg PR. Impact of drought on poverty in Somalia. World Bank Policy Research Working Paper, (8698); 2019.

[16] Maxwell D, Majid N. Famine in Somalia: competing imperatives, collective failures, 2011–12. Oxford: Oxford University Press; 2016.

[17] Jaspars S, Adan GM, Majid N. Food and power in Somalia: business as usual? A scoping study on the political economy of food following shifts in food assistance and in governance. The London School of Economics and Political Science Conflict Research Programme 2019.

[18] Checchi F, Robinson C. Mortality among populations of southern and central Somalia affected by severe food insecurity and famine during 2010–2012—Somalia. Nairobi: Food and Agriculture Organization; 2013.

