# Supplementary material for: A systems analysis of structures, dynamics, archetypes of the Somalia famine, 2011–2012
Source: J Public Health Policy. 2026 Aug 12;47(Suppl 1):71–81. doi: 10.1057/s41271-026-00658-1 (PMC13503269; doi:10.1057/s41271-026-00658-1)
Supplement: Supplementary file 1 [file 41271_2026_658_MOESM1_ESM.docx]

**Table 1: Reinforcing feedback loops**

| Loop ID | Loop Type | Name of Loop | Key Mechanism | Direction of Effect | System Outcome |
| --- | --- | --- | --- | --- | --- |
| R1 | Reinforcing | Climate–Agriculture–Mortality Loop | Drought → reduced water availability → lower crop/livestock production → reduced food supply → higher malnutrition → higher mortality → reduced labor → further reduced production | Positive feedback | Accelerates food insecurity and mortality through ecological stress and productivity loss |
| R2 | Reinforcing | Global Price Shock & Import Dependency Loop | Rising global food prices → reduced imports → higher local prices → reduced food access → higher malnutrition and mortality→ geopolitical conflict | Positive feedback | Deepens food insecurity through market dependency and affordability shocks |
| R3 | Reinforcing | Health System Collapse Loop | Weak health infrastructure → increased disease incidence → higher mortality → reduced health workforce → further health infrastructure weakening | Positive feedback | Escalates mortality through degraded healthcare capacity |
| R4 | Reinforcing | Migration–Remittance–Food Access Loop | Food access → migration → reduced local social capital → reduced remittances → lower purchasing power → reduced food access | Positive feedback | Intensifies food scarcity by weakening social and financial coping systems |
| R5 | Reinforcing | Conflict–Aid Disruption Loop | Conflict (Al-Shabaab + governance constraints) → aid diversion + restricted mobility → reduced food aid → higher malnutrition and mortality → increased geopolitical conflict | Positive feedback | Weakens humanitarian response and reinforces famine conditions |
| R6 | Reinforcing | Mobility & Access Constraint Loop (Hold Effect) | Rising conflict → reduced mobility + restricted migration → reduced food access → increased malnutrition | Positive feedback | Prevents effective system recovery by limiting response capacity |

**Table 2: Balancing feedback loops**

| Loop ID | Loop Type | Name of Loop | Key Mechanism | Direction of Effect | System Outcome |
| --- | --- | --- | --- | --- | --- |
| B1 | Balancing | Migration and asset liquidation Loop | Reduced food access → migration + livestock sales → purchasing power→ partial reduction in immediate food insecurity | Negative feedback (dampening but weak) | Provides short-term buffering |
| B2 | Balancing | Humanitarian Food Aid Loop (Constrained) | Rising food insecurity → increased food aid → improved food availability → reduced malnutrition and mortality →reduced food insecurity | Negative feedback | Intended stabilizer but heavily constrained by Al-Shabab conflict and OFAC restrictions |
| B3 | Balancing | Informal Financial Support Loop (Hawala & Remittances) | Migration → diaspora remittances + cash transfers → increased purchasing power → improved food access → reduced malnutrition | Negative feedback (partial stabilizer) | Moderately stabilizes food access through informal financial systems |
| B4 | Balancing | Environmental & Market Recovery Loop | Improved rainfall + lower global food prices increases food supply→ →lower local food price → increased food access → reduced malnutrition and mortality → reduced food insecurity | Negative feedback (exogenous stabilizer) | External stabilizing force |
